# Comparison of FRAX Scores of Southern California Females of Mexican Descent Using US Hispanic and Mexico Database

**DOI:** 10.4061/2011/531359

**Published:** 2011-05-25

**Authors:** Keaton M. Nasser, Augusto Focil, Stuart L. Silverman

**Affiliations:** ^1^Osteoporosis Medical Center, 8641 Wilshire Boulevard, Suite 301, Beverly Hills, CA 90211, USA; ^2^California Hispanic Osteoporosis Foundation, Oxnard, CA 93030, USA; ^3^Cedars-Sinai/UCLA, Los Angeles, CA 90048, USA

## Abstract

*Introduction*. Regional differences for fracture risk of US Hispanics may vary by national origin. In California, where a majority of the Hispanic population is of Mexican descent, it is of interest to compare the FRAX absolute risk using the US Hispanic and Mexico databases. *Methods*. We collected FRAX risk factor data from 134 women of Mexican descent in southern California. The FRAX risk score was calculated using the US Hispanic and Mexican databases, using the NOF guidelines for osteoporosis to compare the number of patients that would be selected for treatment. *Results*. The 10-year absolute risk of major osteoporotic fracture among women of Mexican descent using the US Hispanic database was 4.82 ± 5.03 (95% CI 3.97–5.67) compared to 4.86 ± 4.72 (CI 3.98–5.44) using the Mexico database (*P* = .94). The 10-year risk for hip fracture was 0.86 ± 1.78 (CI .56–1.16) compared to 1.12 ± 1.97 (CI .79–1.45, *P* = .26). The mean conformity for meeting the interventional threshold by either risk score was 94.8%. *Conclusion*. The comparison between the FRAX databases demonstrates a similarity in the absolute risk of major osteoporotic fracture. Differences are noted in the absolute number of hip fracture subjects at risk, but there is a high rate of conformity.

## 1. Introduction

The FRAX calculator for the United States is unique in that there exist distinct databases for ethnic minorities. However, the addition of ethnic databases may not fully distinguish the variability of racial, ethnic, and national origins of the Hispanic community in the Unites States. There are regional differences in hip fracture incidence for Hispanics within the US [1]. For example, moving from the east coast to the west coast, the fracture rates for Hispanics increase as much as three-fold [1, 2]. Even though the US Hispanic FRAX database was developed based on NHANES 3 BMD data with oversampling for Mexican Americans [3], it may underestimate the fracture risk for Californian Hispanics, most of whom are of Mexican descent. Clinically, this could lead to the potential undertreatment of high-risk individuals.

A Mexico database was recently added to FRAX. Unlike the US Hispanic database, which relies solely on BMD data to estimate risk, the Mexico database is more representative of the broader Mexican population and is based on observed prevalence and incident rates of actual fracture events [4]. In California, a majority of the Hispanic population is of Mexican descent: of the nearly 13.5 million Hispanic residents, 84% (39% foreign born) are of Mexican descent [5]. The similarities between Mexican and California Hispanics are supported by the age-adjusted hip fracture incidence rates. Incidence rates per 1,000 person-years were 2.03 [4] for Mexican females compared to 1.97 for California Hispanics [6]. The data suggest that there is similar risk for fracture between Mexicans and California Hispanic females that might not be represented nationally by the US Hispanic FRAX database. It is not known if FRAX estimates for Hispanic women in Southern California differ between the use of either the US Hispanic database or the Mexico database. 

Our objective was to compare the number of Southern Californian, Mexican-American (of Mexican descent) women who met the NOF treatment threshold using the FRAX US Hispanic and Mexico databases.

## 2. Methods

FRAX clinical risk factor data, without BMD results, was collected from patients in Southern California [7]. Subjects were recruited at health fairs and conferences in Burbank, Los Angeles, Santa Barbara County, and Ventura County. Subjects were postmenopausal, Hispanic women greater than age 40 with no history of using osteoporosis medications (per FRAX guidelines). 160 surveys were completed, of which, 134 were by women self-identified as Hispanic and of Mexican descent.

 The FRAX risk score was calculated using the US Hispanic and Mexican databases. Conformity between the databases was determined by comparing the number of instances where the risk of fracture matched to the instances where risk did not match between databases. In the US, NOF guidelines suggest persons with low bone mass and an FRAX score above 3% risk of hip fracture or 20% risk of osteoporotic fracture are at high risk and should be considered for treatment [8].

## 3. Results

Table 1 provides the summary of demographics and clinical risk factor data acquired for all subjects of Mexican descent. Subjects of Mexican descent comprised 83.8% (134 of 160) of those surveyed. The breakdown of US native-born and generational data is unknown. Using the US Hispanic FRAX database the 10-year absolute risk of major osteoporotic fracture was determined to be 4.82 ± 5.03 (95% confidence interval (CI) 3.97–5.67) and 10-year risk of hip fracture 0.86 ± 1.78 (CI  .56–1.12). Using the Mexico database, the 10-year absolute risk of major osteoporotic fracture and risk of hip fracture was 4.86 ± 4.72 (CI 4.06–5.66) and 1.12 ± 1.97 (CI  .79–1.45), respectively. The differences in averages between the two databases for major osteoporotic fracture (*P* = .9392) and hip fracture (*P* = .2595) are not statistically significant.

US NOF guidelines for osteoporosis suggest treatment for patients with a BMD less than 1.0 and with a 10-year hip fracture probability ≥3% or a 10-year major osteoporotic fracture risk ≥20%. Because BMD data was not collected, we only used the 20%/3% threshold determinant for intervention. Using the US database, 9 subjects were identified at high risk (Table 2). Using the Mexico database, 16 patients were identified at high risk. The difference in absolute risk of fracture was more pronounced based on hip fracture risk. The mean conformity for meeting the interventional threshold by either risk score was 94.8% (Table 3). The mean conformity for meeting the interventional threshold based solely on the 10-year hip fracture risk was 94.8%. The mean conformity for meeting the threshold based solely on the risk of any major osteoporotic fracture was 99.3%.

## 4. Discussion

The data for the US Hispanic database was extrapolated from BMD and risk factor data from NHANES III [9]. The difficulty in using national data lies in the diversity of the Hispanic community in the USA. The ethnic term “Hispanic” has been defined to include an person of Cuban, Mexican, Puerto Rican, South or Central American, or other Spanish culture or origin, regardless of race [10]. However, even though individuals are classified as Hispanic, many Puerto Ricans and Cubans self-identify as African-American, black or Afro-Caribbean. The FRAX database for Hispanic Americans cannot account for the variability of incident fracture rates across the United States. The US Hispanic database, while oversampled for Mexican-Americans, includes data from other Hispanic racial groups. Because of this, Hispanics cannot be considered as a singular group, and treating patients needs to be aligned more closely with race and national origin.

This study has several limitations. Ultimately, it remains unknown which database more accurately reflects the actual risk of fracture for Hispanic women in Southern California. This information can only be determined by observing fractures prospectively. Additionally, the dataset is limited to only 134 subjects. This study needs to be repeated with a larger dataset.

## 5. Conclusion

The comparison between the Mexican and US Hispanic FRAX databases demonstrates a similarity in the absolute risk of major osteoporotic fracture. Differences are noted in the absolute number of hip fracture subjects at risk, but there is a high rate of conformity. Further research is needed to determine the role and generational limitations of using ancestry and country of origin.

##  Disclosures

Dr. A. Focil is a speaker for Genentech and Warner-Chilcott. Dr. S. L. Silverman has received grants from Eli Lilly and Co. and Alliance for Better Bone Health. He has received honoraria from Eli Lilly, Roche Pharmaceuticals, and Pfizer. And he has consulted for Warner Chilcott, Roche Pharmaceuticals, Roche Diagnostics, Novartis, Pfizer, and Eli Lilly and Co. Funding for this study was provided by Procter & Gamble.

## Figures and Tables

**Table 1:** Characteristics of study subjects (*n* = 134).

	Mean
Age	53 ± 10
Height (cm)	158 ± 7
Weight (kg)	72 ± 13
BMI (kg/m^2^)	28.8 ± 5.3

Risk factor	No. (%)

Fragility fracture	37 (27.6)
Parental hip fracture	11 (8.2)
Alcohol	1 (0.7)
Smoker	6 (4.5)
Glucocorticoid use	19 (14.2)
Rheumatoid arthritis	24 (17.9)

**Table 2:** Number of subjects meeting NOF interventional threshold limits.

	US Hispanic database	Mexico database
High fracture risk for any major osteoporotic fracture	4	3
High risk by hip fracture	8	16
High risk by either measure	9	16
Not at high risk by either measure	125	118

**Table 3:** Conformity of risk assessment between US Hispanic and Mexico FRAX databases for women of Mexican descent.

	Any major osteoporotic fracture	Hip fracture	Any and/or hip
Conformity at high fracture risk	3	8	9

Conformity at low fracture risk	130	118	118

High fracture risk by US Hispanic and low by Mexico	1	0	0

High fracture risk by Mexico and low by US Hispanic	0	8	8

Conformity (%)	99.3	94.8	94.8
